# Anethole rich *Clausena heptaphylla* (Roxb.) Wight & Arn., essential oil pharmacology and genotoxic efficiencies

**DOI:** 10.1038/s41598-022-13511-8

**Published:** 2022-06-15

**Authors:** Mohan Lal, Twahira Begum, Roktim Gogoi, Neelav Sarma, Sunita Munda, Sudin Kumar Pandey, Joyashree Baruah, Raghu Tamang, Samarjit Saikia

**Affiliations:** 1 Agrotechnology and Rural Development Division, CSIR-North East Institute of Science and Technology, Jorhat, 785006 Assam India; 2 AcSIR-Academy of Scientific & Innovative Research, Ghaziabad, Uttar Pradesh 201002 India

**Keywords:** Biochemistry, Biological techniques, Cell biology, Chemical biology, Microbiology, Neuroscience, Plant sciences

## Abstract

Anethole, a widely used industrial flavoring agent is majorly sourced from anise and star anise. The present study is aimed to the in-depth pharmacological analysis i.e. anti-diabetic, skin whitening, neurodegenerative disorder inhibitory activities of anethole-rich *Clausena heptaphylla* leaf essential oil (ARCHEO) (88.59%) as revealed by the Gas Chromatography/Mass Spectrometry (GC/MS) analysis and further confirmed by proton nuclear magnetic resonance ^1^H-NMR as well as to compare with standard compound anethole*.* ARCHEO (ABTS EC_50_ 6.97 ± 0.004 µg/mL; Protease assay 4.51 ± 0.004 µg/mL) outperformed the standard compound anethole (ABTS EC_50_ 9.48 ± 0.048 µg/mL; Protease assay EC_50_ 22.64 ± 0.016 µg/mL) in antioxidant and anti-inflammatory experiments. ARCHEO was also shown to be more effective than the reference compound anethole in terms of anti-diabetic activity (EC_50_ 22.35 ± 0.121 µg/mL), tyrosinase inhibitory activity (EC_50_ 16.45 ± 0.012 µg/mL), and anti-cholinesterase activity (EC_50_ 22.32 ± 0.016 µg/mL). However, ARCHEO exhibited lower antimicrobial activity towards all the tested microbes compared to standard compound anethole and as for the MIC, ARCHEO was effective only towards *Salmonella typhimurium* (60 µg/mL)*, Streptococcus mutans* (20 µg/mL)*,* and *Aspergillus fumigatus* (75 µg/mL)*.* ARCHEO (11.11%) and anethole (12.33%) showed no genotoxic effect based on *Allium cepa* assay mitotic index value. Thus, ARCHEO could be a commercially viable and widely available cheaper source of anethole, which has buoyant demand in the field of food flavoring, fragrance, and pharmaceutical industries.

## Introduction

Essential oils are natural, concentrated hydrophobic fragranced volatile oily liquid with mixtures of compounds produced by aromatic plants as secondary metabolites^1^. Traditionally essential oils were majorly used as food preservative agents, perfuming and flavoring agents^2–4^. Previously essential oils were lesser employed for their pharmacological properties. However, recent advances have bought the limelight on the pharmaceutical aspects of essential oil. Owing to being natural, in turn being lesser toxic than the synthetic variants essential oils are sought as alternative medicine in recent researches as more people are heading towards natural sources for various treatments^5^. Thus, the need for natural products is on the higher side by the virtue of the trend of the population to move to greener sources.

One of the compounds naturally sourced from essential oil is “anethole” and it’s a derivative of alkoxy propenyl benzene which occurs naturally in *trans* and *cis* form^6^. Among its isomers, the naturally occurring isomer form of anethole is *trans*-anethole which covers around 90% of naturally found anethole^6^. The *trans*-anethole has a sweeter herbaceous aroma and it tastes sweet which is about ten times sweeter than common edible sugar^7^. It is an important flavoring compound with extensive utilization in the field of food and confectionery, perfumery, cosmetics, and pharmaceutical applications^8,9^. Traditionally, anethole-containing plants were used for treating issues of nervous disturbances, inflammation, gastro-intestinal problem as well as catarrh of the respiratory tract^8^. The anethole containing plants also finds their use as spices, mouth freshener, and sweetener^10^. The anethole is majorly sourced from anise, star anise and fennel^7^. The present study is aimed at the evaluation of anethole-rich *Clausena heptaphylla* essential oil for their different pharmacological activities.

*Clausena heptaphylla* (Roxb.) Wight & Arn., belonging to the family Rutaceae is an aromatic shrub or small tree. The plant is native to India (particularly to the Northeast region), Bangladesh, Laos, Myanmar, Nepal, Thailand, and adjoining regions^11^. The plants belonging to the genus *Clausena* are known to possess various medicinal properties owing to which it finds its use in various traditional treatments for muscular pain, headache, and malarial fever in addition to their use as an insecticide, astringent, diuretic, and vermifuge^12^. The leaves of the plant are reported to possess a pleasant yet strong aroma. The leaves contain the essential oil which on extraction is reported to be light yellow colored^13^.

*Clausena heptaphylla* leaf essential oils are volatile compounds that have a complex mixture^14^. Hence, the assessment of their pharmacological aspects plays a major role in throwing light on their pytotherapeutic role. Essential oils obtained from different plants have been used for antioxidant potential and to treat inflammatory disorders via different mechanisms to reduce inflammation. Therefore, the present investigation has tried to evaluate the antioxidant, inflammation inhibitory capacity as well as toxicity level of the essential oil. Furthermore, the analysis of the essential oil for its antimicrobial, anti-diabetic, tyrosinase inhibitory, anti-cholinesterase activity, and lastly genotoxicity test would pave the way for large-scale application of anethole rich *C. heptaphylla* for their therapeutic application.

Anethole-rich *C. heptaphylla* essential oil would be highly valuable if it can replace the synthetic anethole being safer and highly cost-effective for being of botanical origin. So far, no detailed study has been carried out on anethole-rich *C. heptaphylla* essential oil. To the best of our knowledge, there are only two reports on the Gas Chromatography (GC) analysis of leaf essential oil composition of *C. heptaphylla* from India^13,15^. However, no report on the *in-vitro* biological study is available in the public domain. Essential oils are now widely used in a variety of industries, including pharmaceuticals, flavorings, and perfumery. As a result, determining their potential use in these domains as well as their toxicity is crucial. Therefore, present investigation was designed to evaluate the chemical composition of *C. heptaphylla* leaves essential oil and to compare its *in-vitro* antioxidant, anti-inflammatory, anti-diabetic, skin whitening, neurodegenerative inhibitory, anti-microbial, and genotoxic activities with pure compound anethole.

## Materials and methods

### Chemicals

Ascorbic acid, acetic acid, anethole standard, 2, 2-diphenyl-1-picrylhydrazyl (DPPH), methanol, ethanol, sodium phosphate dibasic, hydrochloric acid (HCl), potassium ferricyanide, sodium phosphate monobasic, tricarboxylic acid (TCA), ferric chloride, ABTS (2, 2′-azino-bis (3-ethylbenzothiazoline-6-sulfonic acid), ferrous chloride, ferrozine, ethyl methanesulphonate (EMS), ethylenediamminetetracetic acid (EDTA) and acetocarmine were procured from Sigma-Aldrich Co. Germany, mueller–hinton broth (MHB) and agar (MBA), potato dextrose broth (PDB) and agar (PDA), casein, perchloric acid, potassium persulfate, ethylene diamine tetra acetic acid, and trypsin were procured from (HiMedia Nashik, India), sodium diclofenac was procured from Geltec Private Limited, Bangalore, acarbose was purchased from Sisco Research Laboratories Pvt. Ltd. (SRL) and other standards of the essential oil were procured from Sigma Aldrich Germany., and albumin was extracted from a fresh egg collected from local market of Jorhat, India.

### Instruments used

Gas Chromatography/Mass Spectrometry (GC/MS) (Agilent Technologies) and Nuclear Magnetic Resonance (NMR) (ADVANCE III FT-NMR Spectrometer (500 MHz), Bruker) was used for the analysis of essential oil. Spectrophotometer (Genesis 10UV Spectrophotometer) and confocal microscope (Model Leica DM3000 LED) were used for the measurement of spectrophotometric reading and observation of the mitotic stages, chromosomal aberrations of onion root cell, respectively.

### Collection and identification of plant material

Fresh leaves of *C. heptaphylla* (*cv*.Jor Lab CH-2) were collected from the experimental farm of CSIR-North East Institute of Science and Technology, Jorhat, Assam (26° 44′10″N; 94° 9′30″E) in September, 2020 and authenticated by plant breeder Dr. Mohan Lal, Senior Scientist, of the Agrotechnology and Rural Development Division, CSIR-NEIST Jorhat. The plant name was confirmed with the Plant List as accessed on September 2020. A voucher specimen has been deposited at the departmental herbarium vide specimen No RRJ CH-01117 and plants were also maintained at the institute field gene bank.

### Essential oil extraction and chemical analysis, identification through GC/MS and NMR

Fresh leaves of *C. heptaphylla* (300 g) were washed and essential oil was extracted using a Clevenger apparatus (4 h, 3 L distilled water). The essential oil extracted was recovered and treated with sodium sulfate anhydrous to remove excess water and stored at 4 °C for further analysis.

### GC/MS analysis

The Gas Chromatograph (Agilent Technologies) was utilized in conjunction with an MSD 5975 C mass selective detector and a fused silica capillary HP-5MS column (30 m × 0.25 mm i.d; 0.25 µm film thickness). At a rate of 1 mL/min, helium gas was used as a carrier. The oven temperature was first set at 40 °C for 2 min, then steadily increased to 250 °C at 5 °C/min, and finally set at 300 °C at 30 °C/min for 30 min. The sample (1 µL) was diluted in acetone, and the diluted sample (1:100, v/v) was injected (split injector, 1:20 for 1 min) while the temperature was kept constant at 250 °C. The GC/MS has a scan range of 45–650 amu. Peaks in total ion chromatogram profiles were detected by comparing mass spectra data to the NIST/Willey mass spectral library, and then confirming using Kovat's index on the HP-5MS column^16^. To generate calibration curves for quantification, representative real chemicals were run using the same GC condition. The retention indices (RI) were calculated using Kovat's method utilizing alkanes (C8–C32) as the benchmark. The essential oil content was confirmed using GC-FID (Thermo Scientific TRACE 1110) coupled to a TG-WAXMS column (60 m × 0.25 µm) and a flame ionization detector. Standard of anethole was run with the same GC conditions. Quantification was done by the area normalization method. The approach provided by Gogoi et al.^17^ was followed for the analysis.

### NMR analysis

Bruker's ADVANCE III FT-NMR Spectrometer (500 MHz) was used to analyze the essential oil of *C. heptaphyla* for NMR spectroscopic study. Dimethyl sulfoxide was used to dissolve the crude ARCHEO. Residual solvent peaks were used as a point of comparison. Data was recorded using the Zg30 conventional pulse programme, and NMR data was analyzed using the Mestre Nova software.

### Antioxidant activity

The antioxidant activity of ARCHEO, as well as anethole (pure compound) was performed by different tests as mentioned below.

### DPPH free radical scavenging activity

The DPPH assay of ARCHEO was performed by the method given by Noumi et al.^18^, with a slight modification using ascorbic acid as standard antioxidant agent.

### ABTS assay

ABTS (2, 2′-azino-bis (3-ethylbenzothiazoline-6-sulfonic acid) assay of ARCHEO was carried out using ascorbic acid as standard following slightly modified protocol of Re et al.^19^.

### Metal chelating activity assay

The antioxidant activity of the ARCHEO, EDTA (standard) and pure compound anethole was also analyzed by using a metal chelating activity assay using as per the protocol described by Dinis et al.^20^.

### Reducing power activity

The reducing power activity of ARCHEO and standard ascorbic acid was calculated according to a slightly modified method of Oyaizu^21^.

### Anti-inflammatory activity

#### Protein egg albumin denaturation assay

The anti-inflammatory activity of ARCHEO, sodium diclofenac (standard) as well as anethole (pure compound), was determined by protein denaturation assay as described by Sangita et al.^22^.

#### Protease inhibitor activity

The protease inhibitor assay of ARCHEO was performed according to Kunitz^23^, with a slight modification using sodium diclofenac as standard anti-inflammatory drug.

#### Antidiabetic activity assay

The antidiabetic activity of ARCHEO, acarbose (standard) and pure compound anethole was analyzed using the standard method as per the protocol described by Xiao et al.^24^.

#### Anti-tyrosinase activity assay

Tyrosinase inhibitory activity of ARCHEO was assayed using a modified dop-achrome method as per the protocol of Sarikurkcu et al.^25^ using kojic acid as standard tyrosinase inhibitor drug.

#### Acetylcholinesterase inhibitory assay

Acetylcholinesterase activity of ARCHEO, galanthamine (standard) and anethole was analyzed using standard methodology of Ellman et al.^26^.

### Antimicrobial activity of leaf essential oil

#### Microbial strains

The antibacterial activity of ARCHEO and standard anethole were performed against the gram-positive bacteria i.e., *Staphylococcus aureus* ATCC-11632, *Bacillus subtilis* ATCC-11774, *B. cereus* ATCC-10876, *Streptococcus mutans* ATCC-25175, and gram-negative bacteria i.e., *Salmonella typhimurium* ATCC-13311. Antifungal activity of the leaf essential oil and standard compound anethole were also performed against *Aspergillus fumigatus* ATCC-204305, *A. niger* ATCC-16885, *Saccharomyces cerevisiae,*ATCC-9763 and *Candida albicans* ATCC-66027. Ciprofloxacin and fluconazole were used as standard antimicrobial drugs at a concentration (10 μg/disc) for bacterial and fungal strains, respectively.

#### Preparation of the inoculums and media

MHA was used for culturing the bacteria from bacterial broth and kept at 37 °C for 24 h. The fungal broth was used to prepare fungal culture in PDB and it was kept at 28 °C for 48 h. The antibacterial and antifungal activities of leaf essential oil of *C. heptaphylla* and standard anethole were performed in MHA and PDA, respectively.

#### Disc diffusion method

The concentrations of ARCHEO and standard anethole were prepared at the ranges (50–500 µg/mL) with methanol and performed by disc diffusion (6 mm) method for both antibacterial and antifungal activities^27^. The method of broth micro dilution was used for evaluating the minimal inhibitory concentration (MIC) of ARCHEO for bacteria and fungi as per standard protocol described by Rafael et al.^28^.

#### *Allium cepa* assay for genotoxicity

Genotoxicity assay of the leaf essential oil of *C. heptaphylla,* as well as anethole (pure compound), was performed to check chromosomal aberration and mitotic index using *Allium cepa* root tips as per protocol of Grant^29^. The percentage of mitotic index (MI) was also calculated from dividing cells of treated EMS, standard anethole and ARCHEO as per described protocol of Sehgal et al.^30^. The frequency of chromosomal aberration was checked following protocol of Babatunde and Bakare^31^.

### Statistical analysis

The statistical analysis was performed using MS Excel for standard deviation (SD). For the IC_50_ i.e. 50% of inhibition was analyzed using MS-Excel and GraphPad Prism 7.04 version software, and EC_50_ was analyzed using XLSTAT software. Every experiment was replicated three times to reduce the chances of experimental errors (when *p* ≤ 0.05 the difference was considered statistically significant).

### Ethics approval and consent to participate

No animal model was used in this study and the plant samples used in the research complies with international guidelines and regulations. Therefore, no ethical approval was required.

## Results

### GC/MS and NMR analysis

**Figure 1:**
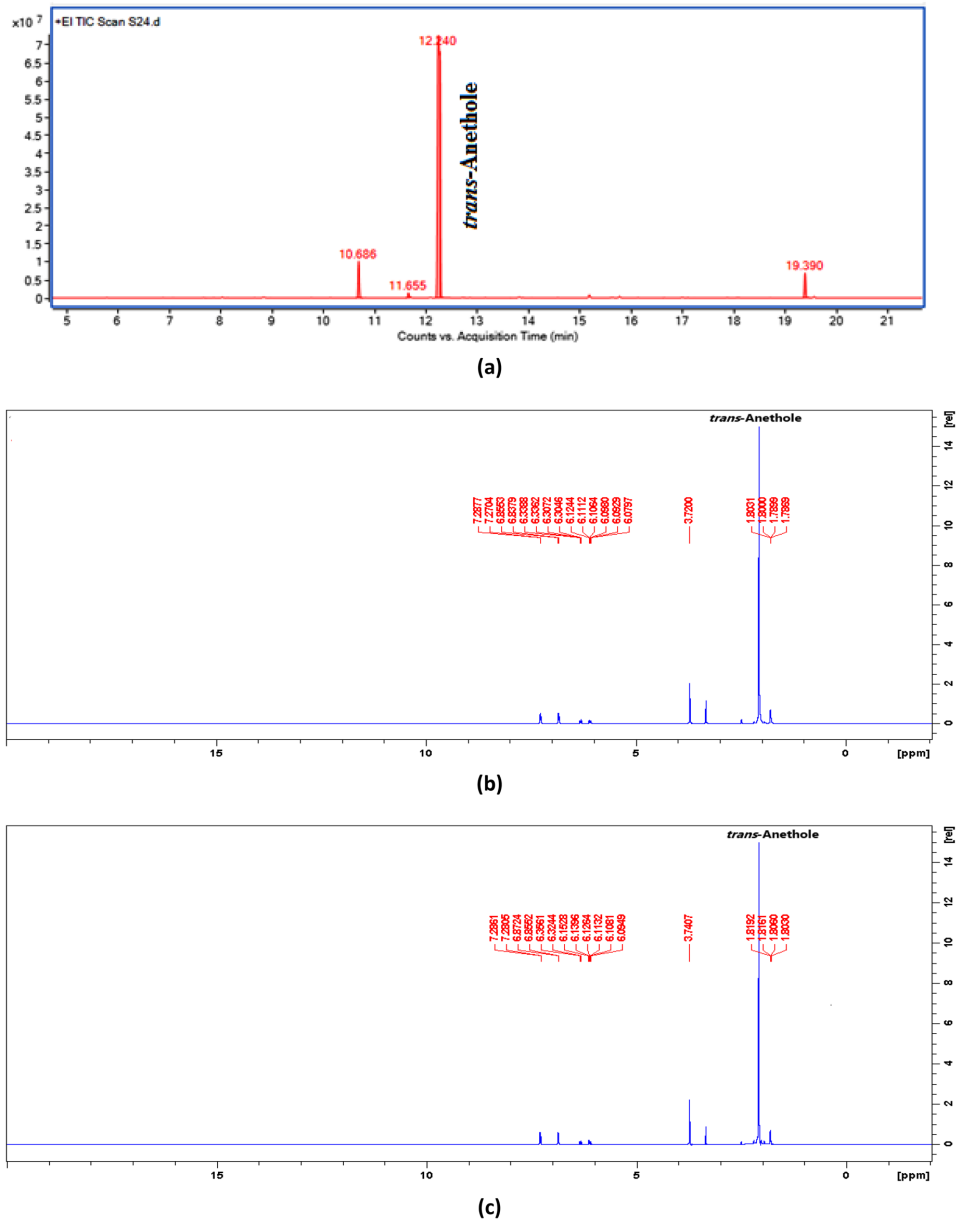
(**a**) Chromatogram of GC/MS analysis of ARCHEO; (**b**) NMR spectra of ARCHEO; (**c**) NMR spectra of the pure standard compound anethole.

Hydro-distillation of leaf *C. heptaphylla* yielded an essential oil (1.22% v/w), which is colorless and found in a solid-state due to the effect of major compound when kept at 4 °C. The GC/MS analysis revealed that the studied ARCHEO is rich in anethole. *Trans-*anethole (88.59%) was found to be the major compound followed by minor compounds estragole (5.36%), ethyl *p* methoxycinnamate (4.03%), and *cis-*anethole (0.80%) was present as trace compound (Table 1, Fig. 1a). The presence of major compound *trans*-anethole in ARCHEO was further confirmed by using NMR analysis. The ^1^H-NMR spectrum of the crude ARCHEO showed and confirmed the presence of the anethole compound in the essential oil. The characteristic peaks were obtained at δ 7.275(d, 2H), 6.85(d, 2H), 6.32(d, 1H), 6.09(m, 1H), 3.72(s, 3H), 1.75(dd, 3H) which corresponds with the ^1^H-NMR spectra of the standard *trans-* anethole (Fig. 1b,c).

**Table 1:** GC/MS analysis of essential oil of anethole-rich *C. heptaphylla* essential oil (ARCHEO).

Sl. no.	Name of the compound	RT	Area %	KI*	KI**	Identification method
1	Estragole	10.68	5.36	1196	1198	1,2,3
2	*cis*-Anethole	11.65	0.80	1254	1256	1,2,3
3	*trans-*Anethole	12.24	88.59	1284	1285	1,2,3
4	*p-*Acetonylanisole	13.88	0.07	1384	1385	1,2
5	Caryophyllene	14.57	0.04	1419	1420	1,2,3
6	*trans-*Ethyl cinnamate	15.18	0.46	1463	1465	1,2,3
7	Benzene, 1,2-dimethoxy-4-(1-propenyl Isoeugenolmethyl ether	15.64	0.10	1492	1492	1,2
8	Bicyclogermacrene	15.77	0.30	1495	1496	1,2
9	Cubebol	16.13	0.03	1515	1518	1,2
10	Ethyl p-methoxycinnamate	19.39	4.03	1773	1774	1,2,3
Total = 100%, Identified compounds = 99.78%, Unidentified compounds = 0.22% Ether (Sl No. 1–3, 7) = 94.85% Acetophenones (Sl No. 4) = 0.07% Sesquiterpene hydrocarbons (Sl No. 5, 8) = 0.34% Oxygenated sesquiterpene (Sl No. 9) = 0.03% Cinnamic acid esters (Sl No. 6, 10) = 4.49%						

KI* Kovats Index Literature^15^, KI** Kovats Index Experimental, 1. Comparison of retention indices with literatures, 2. Comparison of the mass spectra with the mass libraries, 3. Comparing retention time with standards injected with same GC condition.

### Antioxidant activity

Antioxidant activity of ARCHEO and anethole were compared with standard, ascorbic acid to estimate the free radical scavenging power. The IC_50_ value of ascorbic acid, anethole standard, and ARCHEO were calculated and found to be 17.27 μg/mL, 10.94 μg/mL, and 10.01 μg/mL, respectively for DPPH assay as analyzed by MS-EXCEL and 0.60, 0.58, and 0.42 μg/mL respectively as analyzed by Graph Pad Prism. While the EC_50_ values for the DPPH assay as analyzed by XLSTAT are 8.64, 8.69, and 17.55 μg/mL for ARCHEO, anethole and ascorbic acid respectively (Tables 2, 3 and 4). The ABTS assay was also performed for the estimation of the antioxidant potential. The antioxidant potential was confirmed by the IC_50_ values revealing 7.14, 9.26, and 17.61 μg/mL for ARCHEO, anethole, and ascorbic acid respectively as analyzed by MS-EXCEL and 6.27, 34.90, and 39.31 μg/mL respectively as analyzed by Graph Pad Prism. The EC_50_ values for ARCHEO, anethole and ascorbic acid were 6.97, 9.48, and 19.54 μg/mL respectively as per XLSTAT analysis (Tables 2, 3 and 4). For the metal chelating assay, the IC_50_ values for ARCHEO, anethole and ascorbic acid were 21.24, 31.02, and 29.92 μg/mL respectively as analyzed by MS-EXCEL and 5.94, 12.15, and 12.11 μg/mL respectively as analyzed by Graph Pad Prism. The EC_50_ values for ARCHEO, anethole and ascorbic acid were 16.91, 29.54, and 29.33 μg/mL respectively as per XLSTAT analysis (Tables 2, 3 and 4). The comparative studies depicted that the IC_50_ and EC_50_ values of ARCHEO had the strongest capacity among the studied standard anethole and ascorbic acid.

**Table 2:** 50% Inhibition concentrations (IC_50_) determination values for pharmacological activities of ARCHEO and standards using MS-EXCEL software.

Essential oil/standard	DPPH scavenging (µg/mL)	ABTS scavenging (µg/mL)	Metal chelating (µg/mL)	Protein denaturation (µg/mL)	Protease inhibitory (µg/mL)	Tyrosinase inhibitory (µg/mL)	Acetylcholinesterase inhibitory (µg/mL)	α-Amylase inhibitory (µg/mL)
ARCHEO	10.01 ± 0.012	7.14 ± 0.086	21.24 ± 0.021	21.19 ± 0.006	5.05 ± 0.003	17.52 ± 0.012	22.85 ± 0.024	22.80 ± 0.057
Anethole standard	10.94 ± 0.026	9.26 ± 0.002	31.02 ± 0.018	19.26 ± 0.028	20.84 ± 0.042	15.97 ± 0.022	33.81 ± 0.016	22.16 ± 0.12
Ascorbic acid	17.27 ± 0.004	17.61 ± 0.012	ND	ND	ND	ND	ND	ND
EDTA	ND	ND	29.92 ± 0.002	ND	ND	ND	ND	ND
Sodium diclofenac	ND	ND	ND	25.35 ± 0.042	24.54 ± 0.011	ND	ND	ND
Kojic acid	ND	ND	ND	ND	ND	21.12 ± 0.018	ND	ND
Galanthamine hydrobromide	ND	ND	ND	ND	ND	ND	27.62 ± 0.002	ND
Acarbose	ND	ND	ND	ND	ND	ND	ND	23.76 ± 0.014

*ARCHEO* anethole rich *C. heptaphylla* essential oil, *ND* not determined.

**Table 3:** 50% Effective concentrations (EC_50_) determination values for pharmacological activities of ARCHEO and standards using XLSTAT software.

Essential oil/standard	DPPH scavenging (µg/mL)	ABTS scavenging (µg/mL)	Metal chelating (µg/mL)	Protein denaturation (µg/mL)	Protease inhibitory (µg/mL)	Tyrosinase inhibitory (µg/mL)	Acetylcholinesterase inhibitory (µg/mL)	α-Amylase inhibitory (µg/mL)
ARCHEO	8.64 ± 0.12	6.97 ± 0.004	16.91 ± 0.084	24.37 ± 0.042	4.51 ± 0.004	16.45 ± 0.012	22.32 ± 0.016	22.35 ± 0.121
Anethole standard	8.69 ± 0.058	9.48 ± 0.048	29.54 ± 0.032	19.63 ± 0.002	22.64 ± 0.016	15.46 ± 0.004	30.90 ± 0.021	21.75 ± 0.008
Ascorbic acid	17.55 ± 0.056	19.54 ± 0.121	ND	ND	ND	ND	ND	ND
EDTA	ND	ND	29.33 ± 0.064	ND	ND	ND	ND	ND
Sodium diclofenac	ND	ND	ND	27.38 ± 0.012	25.42 ± 0.002	ND	ND	ND
Kojic acid	ND	ND	ND	ND	ND	19.03 ± 0.082	ND	ND
Galanthamine hydrobromide	ND	ND	ND	ND	ND	ND	26.56 ± 0.016	ND
Acarbose	ND	ND	ND	ND	ND	ND	ND	23.52 ± 0.056

*ARCHEO* anethole rich *C. heptaphylla* essential oil, *ND* not determined.

**Table 4:** 50% Inhibition concentrations (IC_50_) determination values for pharmacological activities of ARCHEO, and standards using Graph Pad Prism software.

Eeesential oil/ Standard	DPPH scavenging (µg/mL)	ABTS scavenging (µg/mL)	Metal chelating (µg/mL)	Protein denaturation (µg/mL)	Protease inhibitory (µg/mL)	Tyrosinase inhibitory (µg/mL)	Acetylcholinesterase inhibitory (µg/mL)	α-Amylase inhibitory (µg/mL)
ARCHEO	0.42 ± 0.056	6.27 ± 0.024	5.94 ± 0.014	207.30 ± 0.001	0.28 ± 0.014	48.91 ± 0.086	9.29 ± 0.036	19.57 ± 0.014
Anethole standard	0.58 ± 0.048	34.9 ± 0.004	12.15 ± 0.032	204.80 ± 0.042	0.36 ± 0.012	22.88 ± 0.008	21.42 ± 0.082	13.08 ± 0.016
Ascorbic acid	0.60 ± 0.121	39.31 ± 0.84	ND	ND	ND	ND	ND	ND
EDTA	ND	ND	12.11 ± 0.002	ND	ND	ND	ND	ND
Sodium diclofenac	ND	ND	ND	216.80 ± 0.016	0.42 ± 0.002	ND	ND	ND
Kojic acid	ND	ND	ND	ND	ND	51.56 ± 0.001	ND	ND
Galanthamine hydrobromide	ND	ND	ND	ND	ND	ND	11.61 ± 0.016	ND
Acarbose	ND	ND	ND	ND	ND	ND	ND	20.35 ± 0.002

*ARCHEO* anethole rich *C. heptaphylla* essential oil, *ND* not determined.

**Figure 2:**
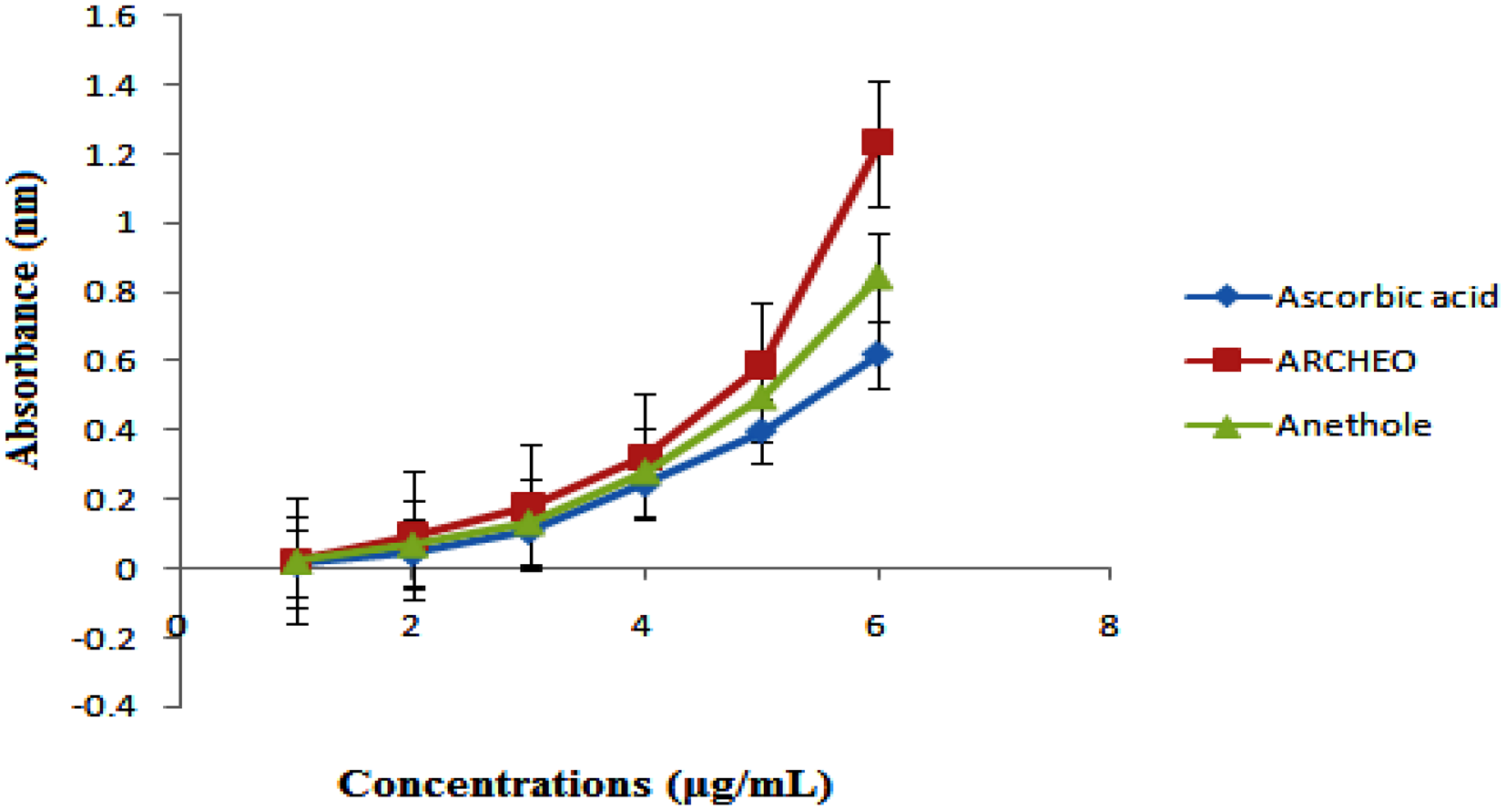
Reducing power activities of ARCHEO*,* anethole and ascorbic acid with standard error bars.

The reducing power of ARCHEO was further used to measure their antioxidant capability. ARCHEO was found to have the highest absorbance which is higher than standard ascorbic acid, and anethole in the same concentration which revealed high antioxidant potential. ARCHEO showed dose-dependent antioxidant activity with respect to ascorbic acid and standard anethole (Fig. 2).

### Anti-inflammatory activity

Anti-inflammatory activity was examined for the potentiality of the ARCHEO with standard compound, anethole, and anti-inflammatory drug, sodium diclofenac. It was observed in protein denaturation assay that ARCHEO and standard anethole showed significantly higher activity than that of sodium diclofenac. The IC_50_ value of standard sodium diclofenac (IC_50_ = 25.35 μg/mL), anethole (IC_50_ = 19.26 μg/mL) and ARCHEO (IC_50_ = 21.19 μg/mL) revealed strongest activity by anethole followed by ARCHEO and sodium diclofenac as analyzed by MS-EXCEL. A similar trend was revealed by EC_50_ values of 24.37, 19.63, and 27.38 µg/mL for ARCHEO, anethole and sodium diclofenac respectively as per XLSTAT analysis. While the IC_50_ values for ARCHEO, anethole, and sodium diclofenac as per Graph Pad Prism analysis was 207.31, 204.82, and 216.86 µg/mL respectively (Tables 2, 3 and 4).

ARCHEO and standard anethole also showed anti-inflammatory activity in protease inhibitor assay. The IC_50_ value of ARCHEO showed stronger anti-inflammatory activity than that of anethole and sodium diclofenac with values of 5.05, 20.84, and 24.54 µg/mL respectively as per MS-EXCEL analysis. As per XLSTAT analysis EC_50_ values for ARCHEO, anethole, and sodium diclofenac were 4.51, 22.64, and 25.42 µg/mL respectively. Lastly, the IC_50_ values for ARCHEO, anethole, and sodium diclofenac were 0.28, 0.36, and 0.42 µg/mL respectively as per Graph Pad Prism analysis (Tables 2, 3 and 4).

### Anti-diabetic activity

ARCHEO was analyzed for its anti-diabetic activities, which revealed strong amylase inhibitory effects of ARCHEO were better than standard acarbose. The inhibitory effects of pure compound anethole were slightly better than both EO and acarbose. The IC_50_ values were 23.76, 22.80, and 22.16 µg/mL for acarbose, ARCHEO and pure compound anethole respectively as analyzed by MS-EXCEL and 20.35, 19.57 and 13.08 µg/mL respectively as analyzed by Graph Pad Prism. XLSTAT analysis revealed EC_50_ values for ARCHEO, anethole and acarbose were 22.35, 21.75, and 23.52 µg/mL respectively. The IC_50_ revealed values of (Table 2, 3 and 4).

### Anti-tyrosinase activity

The skin whitening capacity of ARCHEO and pure anethole was analyzed using tyrosinase inhibitory activity assay. The activity of anethole and essential oil confirmed from the IC_50_ value 15.97, 17.52 µg/mL respectively; which was way better than standard kojic acid 21.12 µg/mL as analyzed by MS-EXCEL. The EC_50_ was also in a similar line with values of 16.45, 15.46, and 19.03 µg/mL for ARCHEO, anethole, and kojic acid respectively as per XLSTAT analysis. The IC_50_ as analyzed by Graph Pad Prism revealed values of 48.91, 22.88, and 51.56 µg/mL for ARCHEO, anethole, and kojic acid respectively (Table 6).

### Anti-cholinesterase activity

Neurodegenerative disorder inhibitory activity was analyzed using acetyl cholinesterase inhibitory assay. The ability of neurodegenerative inhibitory effect was confirmed from the EC_50_ value as analyzed by XLSTAT software which was 22.32, 30.90, and 26.56 µg/mL for ARCHEO, pure anethole, and galanthamine respectively. While the IC_50_ exhibited were 11.61, 9.29, and 21.42 µg/mL by galanthamine, ARCHEO and pure anethole respectively, as analyzed by Graph Pad Prism. Lastly, the IC_50_ as analyzed by MS-EXCEL revealed values of 22.85, 33.81 and 27.62 µg/mL for ARCHEO, anethole and galanthamine respectively (Tables 2, 3 and 4).

### Antimicrobial activity

Disc diffusion and MIC method were employed to ARCHEO and standard anethole for evaluation of their antimicrobial activities. The antimicrobial test revealed that standard anethole showed better antibacterial activity against all the tested microbes as compared to ARCHEO (Table 5).The antimicrobial activity was found to be dose-dependent with the highest at 500 μg/mL for both anethole standard and ARCHEO. However, ARCHEO showed a significantly lower zone of inhibition for all the tested microbes as compared to anethole (Table 5). The MIC results revealed ARCHEO was effective only against *S. typhimurium, S.mutans* and *A. fumigatus.* While standard anethole was effective against *S. aureus, B. subtilis, S. typhimurium, S.mutans,* and *A. fumigatus* (Table 5)*.* However, ciprofloxacin and fluconazole were effective against all the tested microbes which reveal the antimicrobial property of ARCHEO.

**Table 5:** Zone of inhibitons and minimal inhibitory concentration (MIC) for ARCHEO and anethole standard against different bacterial and fungal strains.

Microorganisms	50 (μg/mL) mm		100 (μg/mL) mm		250 (μg/mL) mm		500 (μg/mL) mm		ARCHEO MIC (μg/mL)	AS MIC (μg/mL)	(ciprofloxacin/fluconazole) (10 µg/disc)
	ARCHEO	AS	ARCH	AS	ARCH	AS	ARCH	AS			
*S. aureus*	–	–	–	8	–	10	11	14	NA	95	19 ± 0.017
*B. subtilis*	–	–	7	8	9	11	11	13	NA	90	16 ± 0.015
*B. cereus*	–	–	–	–	12	13	14	16	NA	NA	15 ± 0.013
*S. typhimurium*	–	8	10	12	11	15	14	17	60	45	21 ± 0.015
*S.mutans*	11	12	14	16	17	18	20	23	20	15	7 ± 0.015
*A. fumigatus*	–	–	8	12	10	14	13	18	75	65	16 ± 0.011
*A. niger*	–	–	–	–	–	16	18	21	NA	NA	12 ± 0.013
*S. cerevisiae*	–	–	–	–	–	–	12	16	NA	NA	14 ± 0.016
*C. albicans*	–	–	–	–	8	15	11	19	NA	NA	20 ± 0.014

*ARCHEO* anethole rich *C. heptaphylla* essential oil, *AS* anethole standard, *NA* not applicable.

### *Allium cepa* assay for genotoxicity test

*Allium cepa* test was performed to measure the growth of the root after the treatment of ARCHEO, standard anethole, and EMS at a concentration of 1 μL/mL. After 72 h, significantly ARCHEO and anethole have no inhibitory effect on root tips was observed as compared to negative control but treated EMS was observed to be highly effective on the growth of root tips. The treated root length of *A. cepa* was found to be 0.88 ± 0.010, 0.61 ± 0.011, 0.65 ± 0.017, and 0.07 ± 0.012 cm in distilled water, ARCHEO, standard anethole, and EMS respectively. During the growth of onion root, it was observed that the anethole and ARCHEO concentration did not find any prevention of root growth as compared to the negative control, distilled water (Table 6). No toxic effect of ARCHEO and standard anethole were observed on the growth of the root of *A.cepa* as compared to EMS*.*

**Table 6:** Root lengths of *Allium cepa* after treatment of ARCHEO*,* anethole (standard) and EMS.

Concentrations (μL/mL)	Before treatment (in cm) ± SD	After treatment (in cm) ± SD	Root length on 72 h (in cm) ± SD
Distilled water	7.54 ± 0.010	8.42 ± 0.012	0.88 ± 0.010
EMS	7.24 ± 0.014	7.31 ± 0.013	0.07 ± 0.012
ARCHEO	7.56 ± 0.013	8.17 ± 0.011	0.61 ± 0.011
Anethole standard	7.71 ± 0.016	8.36 ± 0.009	0.65 ± 0.017

*ARCHEO* anethole rich *C. heptaphylla* essential oil, *EMS* Ethyl methanesulphonate.

The mitotic index (MI) of ARCHEO and standard anethole was calculated and compared with positive and negative control. MI of ARCHEO was 11.11% which was less than distilled water 14.73%, as it compared to the positive control, EMS 1.96% at 1μL/mL concentration. A comparative study of MI showed that anethole (12.33%) has higher MI than ARCHEO but less than the negative control. Although MI of anethole was higher than that of ARCHEO, values were almost in the same ranges, which signified the similar efficacy on onion roots (Table 7). The percentage of MI of ARCHEO and anethole indicated that it has no toxic effect on the growth of onion roots as compared to both the controls. At the same concentration, dividing cells numbers were calculated at different stages, in treated ARCHEO and anethole were found in prophase (64.44%; 59.46%), metaphase (20.00%; 18.92%), anaphase (6.67%; 13.51%), and telophase (8.89%; 8.10%) whereas in case of treated EMS, prophase (86.73%), and metaphase (13.27%), but no cells were found in anaphase and telophase stages. It was observed that both ARCHEO and anethole have almost similar activity to the negative control, where no toxic effect was found on roots (Tables 7 and 8).

**Table 7:** Mitotic index and different stages of dividing cells in root tips of *Allium cepa* treated with ARCHEO*,* anethole (standard) and EMS.

Concentration (μL/mL)	Mitotic index (%)	Prophase (%)	Metaphase (%)	Anaphase (%)	Telophase (%)
Distilled water	14.73	48.65	33.27	12.42	5.66
EMS	1.96	86.73	13.27	0	0
ARCHEO	11.11	64.44	20.00	6.67	8.89
Anethole standard	12.33	59.46	18.92	13.51	8.10

*ARCHEO* anethole rich *C. heptaphylla* essential oil, *EMS* ethyl methanesulphonate.

**Table 8:** Chromosomal aberration test for ARCHEO, anethole (standard) and EMS.

Compound	Concentration	Time	Bridges	Stickiness	Clumped	Multipolarity	Breakage	Total abberations
Distilled water	01.00 µL/mL	72 h	8	9	2	5	6	06.00%
ARCHEO			14	11	6	10	8	09.80%
Anethole standard			12	4	7	3	9	07.00%
EMS			30	28	14	21	15	21.60%

*ARCHEO* anethole rich *C. heptaphylla* essential oil, *EMS* ethyl methanesulphonate.

**Figure 3:**
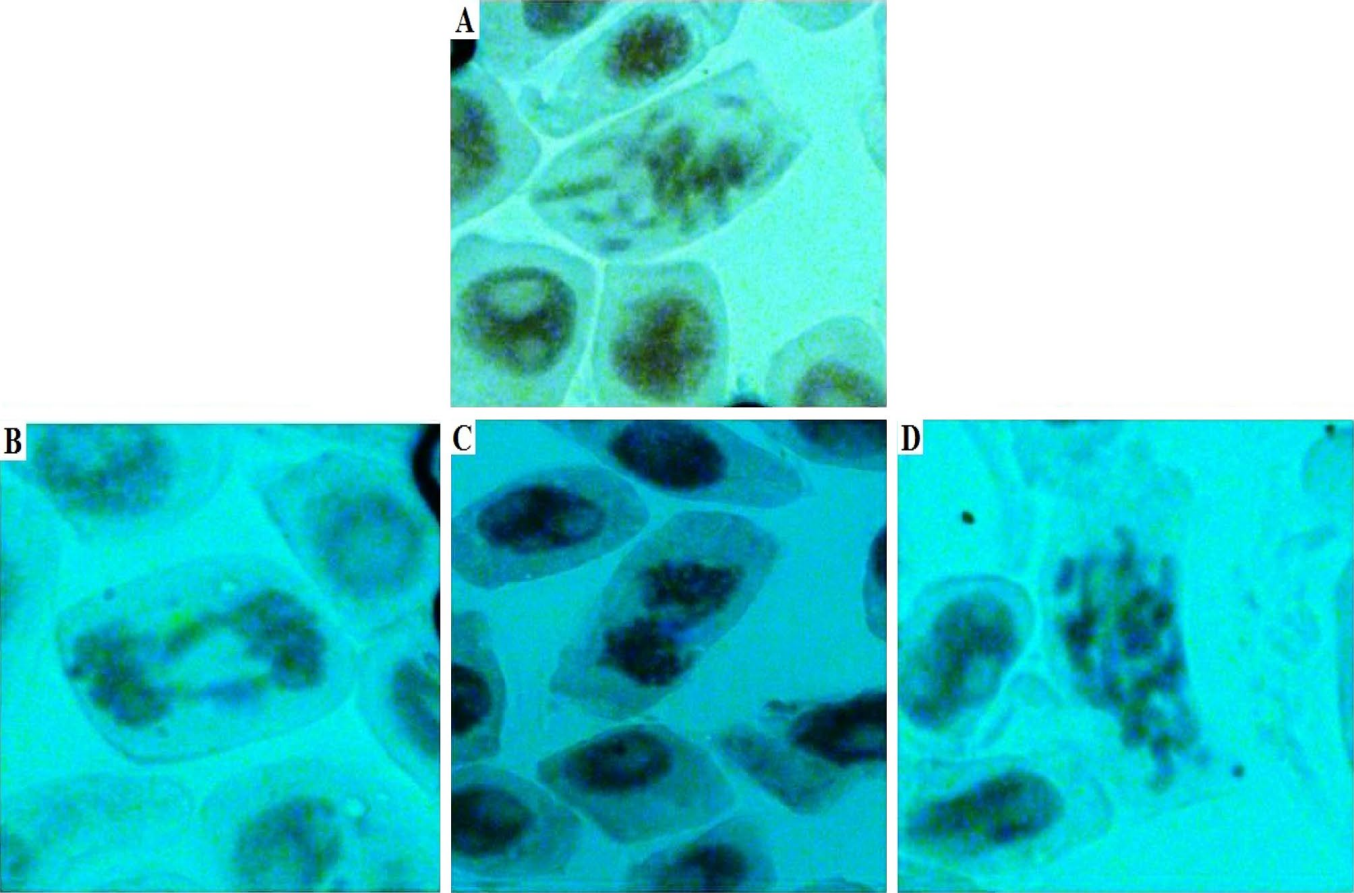
Chromosome aberrations (*Allium cepa* assay, genotoxicity); (**A**) Chromosome break, (**B**) Chromosome bridge, (**C**) Chromosome clump and (**D**) Stickiness chromosome.

The chromosomal aberration of *A. cepa* root tips was observed for the assessment of *in-vitro* chromosomal damage. In the assessment, the chromosome aberrations, bridges, clumps, and stickiness were checked after 72 h of the treatment of ARCHEO*,* standard anethole, and EMS. As treated roots of both essential oil and anethole were compared with treated distilled water and EMS, it was distinctly showed that no chromosomal aberration was observed (Table 8 and Fig. 3).

## Discussions

So far, there are only two studies reported on the GC analysis of *C. heptaphylla* essential oil composition from Northeast India. One of the reports by Nath et al.^15^, revealed that in *C. heptaphylla* essential oil anethole was present in both stages of flowering and fruiting of the leaf (98.2%) and fruit (61.67%) essential oil. Study by Ahmad et al.^13^ revealed *trans*-anethole constituted 92.6% of the essential oil composition of *C. heptaphylla* from Assam. The results of the present study were in similar line to previous reports. Although very few numbers of compounds was identified in leaf essential oil by Nath et al.^15^, they are important based on use in food, cosmetic and commercial industries. Anethole present in leaf essential oil is ten times sweeter than sugar, which is used as a flavoring substance and also used in oral hygiene products, alcoholic drinks, confectionery applications^7,8^. Anisyldithiolthione, anethole dithione,and anethole trithione are the derivatives drugs of anethole and estragole (9.53%) which is the isomer of anethole are used in pharmaceutical industries^32^. Therefore, the present analysis revealed anethole-rich *C. heptaphylla* essential oil (ARCHEO) composition possessing great potential in pharmaceutical and industrial applications.

One of the previous studies, on *C. heptaphylla* different part extracts revealed standard ascorbic acid has a stronger activity than the alcoholic extracts. The stem bark ethanolic extract showed dose-dependent activity with respect to standard, ascorbic acid^12^. Report on DPPH assay revealed highest scavenging activity was 98.64% for ethanolic extract whereas ascorbic acid showed 99.65% scavenging activity at 1000 μg/mL. The IC_50_ value of the stem bark ethanolic extract was 3.11 μg/mL while that of ascorbic acid was 5.15 μg/mL. The reducing power of stem bark ethanolic extract was 0.73 while for ascorbic acid it was 0.85 at 100 μg/mL^12^ which may be attributed to the presence of terpenes and phenolic compounds exerting its action via free radical scavenging ability^33^. The present investigation supports the aforementioned report. As compared to our studies, the EC_50_ and IC_50_ values reveal that the stem bark ethanolic extract has less activity than that of ARCHEO which may be due to the presence of *trans*-anethole as the major compound present in ARCHEO. The ARCHEO depicted an efficacious correlation between DPPH, ABTS, metal chelating, and reducing power assay which contributed to the antioxidant activity.

ARCHEO and standard anethole also showed anti-inflammatory activity in protease inhibitor assay. Comparative studies of both the assays showed that all the concentration of essential oil has a dose-dependent percent inhibition. It can be hypothesized that pre-treatment of anethole can lead to the reduction of cell numbers of pro-inflammatory macrophages and neutrophils as well as pro-inflammatory mediators^34^. Moreover, in a previous study of anethole in the pain model reported to reduce in the secretion of inflammatory mediators^35^. The anethole was also found to have an inhibitory effect on the production of NO and PGE2 in regulating non-immune acute inflammation-causing diseases^36^. Apart from the compound anethole there is no earlier report available regarding inflammation inhibitory activity of ARCHEO. There is one closely related species *Clausena harmandiana,* reported for uses as pain reliever. But a study by Wangboonskul et al.^37^ reported, in carrageenan-induced rat root bark ethanolic extract of *C. harmandiana* showed no anti-inflammatory activity. In the HPLC analysis of the extracts showed the presence of dentain 1.71% and nordentain 2.57%. Hence, there was no anethole detected resulting no inflammation inhibitory activities. In the present investigation ARCHEO exhibited a significant inflammation inhibitory activity which could be positively correlated with the presence of anethole in such a huge quantity 88.59%. Therefore, ARCHEO has a strong potential for inflammation blockage due to the presence of anethole and other compounds which could be further utilize for formulation of inflammation inhibitory drug preparation.

The antidiabetic effects of the essential oil in the present study could be directly correlated with the antidiabetic effect of anethole. Anethole has proven antidiabetic activity^8^. According to another report, *trans*-anethole possesses antidiabetic activity which when injected onto diabetic rat showed a significant reduction in the plasma report^38^. Liver the largest organ of human body is the central metabolic organ. This organ plays important role in glucose homeostasis finally regulating blood glucose level^39^. Now the compound anethole being reported several times that it possess hepatoprotective effects which makes the compound a perfect candidate for down regulation of diabetic conditions. One of such study on streptozotocin induced liver injury in rats was reported to protect the liver against diabetic induced hepatic injury upon *trans-*anethole treatment^40^. According to the report, the mechanism behind being *trans-*anethole as hepatoprotective agent is due to its hypoglycemic and antioxidative effects. However, there was not a single scientific report available in the public domain regarding the comparative study of anti-diabetic activity of ARCHEO and pure compound anethole. Anethole itself is a bioactive compound with immense industrial as well as pharmaceutical demand. The present article thereby provides an alternative source with strong evidence of antidiabetic potential for future deep clinical trials.

Anethole being the strongest among the three tested has evidence that its derivatives were already reported for skin protection activities^41^. The strong skin whitening potential of ARCHEO is better than standard kojic acid due to the major compound anethole with such a high quantity. Earlier a study by Nam et al.^42^ reported that *trans*-anethole isolated from *Foeniculum vulgare* inhibited UV-induced melanogenesis by inhibiting ORAI1 activity. According to the report, *trans*-anethole could be a novel approach towards prevention and treatment of UV-induced melanogenesis. There were some recent publications regarding natural products in management of aging ailments^43,44^. But so far there is no such report available regarding *C. heptaphylla* extract or essential oil. Therefore, the present article gives an alternative source for the extraction of pure major compound anethole with skin whitening ability. *C. heptaphylla* will positively be a cheap, easy, and pharmacologically active source for skin whitening product manufacturing industries.

Although, the highest inhibitory effect was shown by the ARCHEO but there are evidences that pure compound anethole itself is a good neuroprotective agent^8^. In the present investigation, the effect of essential oil may be enhanced by the presence of some minor compounds present with the major compound anethole. ARCHEO roused to be strongest among the tested agents, even better than standard cholinesterase inhibitory agent galanthamine. One of the earlier articles reported that anethole contributed well in *I. verum* extract AChE inhibitory effects^45^. Another study reported anethole can improve the activity of anticholinesterase^46^. There was a report by Menichini et al.^47^ of *Pimpinella anisoides* revealed that an aromatic spice fruit ethanolic extract exhibiting AChE and BChE inhibitory activity with IC_50_ values of 227.50 and 362.10 µg/mL respectively. In their experiment one of the most abundant compound was *trans*-anethole and it exihibited highest AChE and BChE activities with IC_50_ values 134.70 and 209.60 µg/mL than other tested compounds limonene and sabinene. Another study by Todirascu et al.^48^ reported memory deficit can be prevented by the use of *Schinus terebinthifolius* essential oil via its antioxidant potential^48^. In the present investigation, strong AChE inhibitory potential of ARCHEO could be due to its strong antioxidant potentials. From the present investigation, it can be said that anethole possesses AChE inhibitory activity but it is less than our studied ARCHEO and standard galanthamine. From the present investigation, we are providing a source of anethole-rich essential oil-bearing plant with immense potential in the field of pharmaceutical sciences. Deep clinical trials of ARCHEO in search of neurodegenerative inhibitory drugs could be trust-worthy.

A study by Minakshi et al.^49^ reported that naturally occurring anethole as well as standard compound anethole, both inhibited bacterial growth. Another study of leaf alcoholic extract of *C. heptaphylla* found that petroleum ether and hot methanol extracts showed the highest zone of inhibition against *B. cereus* (15 mm; 11 mm), *B. subtilis* (14 mm; 7 mm) and *S. aureus* (15 mm; 13 mm) at 500 μg/disc^50^. Moreover, leaf essential oil has also antifungal activity, the highest zone of inhibition against *S. cerevisiae* (12 mm) at 500 μg/mL concentration whereas in fluconazole (14 mm) but *A. fumigatus* (10 mm), *C. albicans* (9 mm) and *A. niger* (6 mm) were showed the lower zone of inhibitory activity in comparison to fluconazole (16, 20 and 12 mm) at the same concentration (Table 8). A comparative study of anethole with ARCHEO revealed ARCHEO has higher activity than anethole at 500 μg/mL concentrations (Table 5). It was reported that the presence of anethole can inhibit the growth of *A. parasiticus* at concentrations of 100, 200 and 300 μg/mL, but at 400 μg/mL can inhibit completely the growth of *A. parasiticus* with increasing the production of aflatoxin, although the quantity of toxins can be decreased by increasing the concentration of anethole^51^. From the above finding, it is observed that standard anethole has stronger antimicrobial potential than that of ARCHEO. The result may be due to the fact that anethole has greater action as a fumigant as compared to direct contact agent as reported by Padilha et al.^52^. Therefore, ARCHEO does not prove to be a potent source of natural antimicrobial agents.

Previous reports suggested that *trans*-anethole is considered as food grade^8^. It has also been considered as non-carcinogenic, non-genotoxic, and considered as safe by the Expert Panel of the Flavor and Extract Manufacturers Association (FEMA) and Food and Drug Administration (FDA)^9^. Thus, from the present investigation it was found that ARCHEO does not pose any genotoxicity at a concentration of 01.00 µL/mL and can be safely used for commercial purposes and formulation of pharmaceutical agents. However, according to a previous article consumption of 1 to 5 mL of anise oil which is normally considered as rich source of anethole associated with nausea, vomiting, seizures and pulmonary edema in human^53^. Hence, present investigation does not also support direct consumption of ARCHEO.

## Conclusions

Considering the multiple bioactivities of anethole, an anethole-rich plant species would be highly beneficial from the industrial and pharmacological point of view. The present study reveals that ARCHEO has great importance in the field of industrial and pharmaceutical application due to the presence of major compound anethole (88.59%). Taking into account the high anethole content the plant could act as a cheaper source of anethole extraction for commercial purposes. Furthermore, in light of the potent antioxidant activity, anti-inflammatory activity, anti-diabetic, skin whitening activity, and neurodegenerative disorder preventive activity it surpassed all the respective standard compounds considered excluding for its antimicrobial activity. In addition, it did not exhibit any genotoxic effect as well. Thus, it possesses high pharmaceutical properties specially for inflammation-inhibitory, anti-diabetic as well as in skin whitening activities, which could be put to greater use for formulation of drug development and various other industrial applications such as flavoring agents in food, confectionery goods, beverages, and as a masking agents in cosmetics due to its ability to camflourage unpleasant odors. The anethole-rich *C. heptaphylla* would thus prove to be flourishing source of anethole from the commercial point of view.

## Data Availability

All data generated or analysed during this study are included in this article.
